# Tomographic optical imaging of cortical responses after crossing nerve transfer in mice

**DOI:** 10.1371/journal.pone.0193017

**Published:** 2018-02-14

**Authors:** Keiichi Maniwa, Haruyoshi Yamashita, Hiroaki Tsukano, Ryuichi Hishida, Naoto Endo, Minoru Shibata, Katsuei Shibuki

**Affiliations:** 1 Department of Neurophysiology, Brain Research Institute, Niigata University, Niigata, Japan; 2 Department of Orthopedic Surgery, Faculty of Medicine, Niigata University, Niigata, Japan; 3 Department of Plastic Surgery, Faculty of Medicine, Niigata University, Niigata, Japan; Medical Photonics Research Center, Hamamatsu University School of Medicine, JAPAN

## Abstract

To understand the neural mechanisms underlying the therapeutic effects of crossing nerve transfer for brachial plexus injuries in human patients, we investigated the cortical responses after crossing nerve transfer in mice using conventional and tomographic optical imaging. The distal cut ends of the left median and ulnar nerves were connected to the central cut ends of the right median and ulnar nerves with a sciatic nerve graft at 8 weeks of age. Eight weeks after the operation, the responses in the primary somatosensory cortex (S1) elicited by vibratory stimulation applied to the left forepaw were visualized based on activity-dependent flavoprotein fluorescence changes. In untreated mice, the cortical responses to left forepaw stimulation were mainly observed in the right S1. In mice with nerve crossing transfer, cortical responses to left forepaw stimulation were observed in the left S1 together with clear cortical responses in the right S1. We expected that the right S1 responses in the untreated mice were produced by thalamic inputs to layer IV, whereas those in the operated mice were mediated by callosal inputs from the left S1 to layer II/III of the right S1. To confirm this hypothesis, we performed tomographic imaging of flavoprotein fluorescence responses by macroconfocal microscopy. Flavoprotein fluorescence responses in layer IV were dominant compared to those in layer II/III in untreated mice. In contrast, responses in layer II/III were dominant compared to those in layer IV in operated mice. The peak latency of the cortical responses in the operated mice was longer than that in the untreated mice. These results confirmed our expectation that drastic reorganization in the cortical circuits was induced after crossing nerve transfer in mice.

## Introduction

Accidental avulsion of nerve roots in the brachial plexus (BP) from the spinal cord can be repaired by crossing nerve transfer connecting the damaged nerve ending with the healthy BP on the opposite side of the lesion [1–4]. This surgery produces a functional reconstruction of the primary somatosensory cortex (S1) of both hemispheres as well as that of the motor cortex [5–7]. Therefore, functional cortical reorganization is expected to be induced in the S1 after the surgery, especially in the opposite side of the injury. Previously, we reported that the reconstructed activities in the opposite side of the injury were visualized after crossing nerve transfer by transcranial flavoprotein fluorescence imaging [8]. Our results suggested that the reconstructed neural activities in the S1 of the opposite side were produced by the propagation of neural activities from the S1 of the same side to the S1 of the opposite side through the corpus callosum [8]. However, the properties of the reconstructed neural activities in the opposite side are unknown. The somatosensory cortex receives thalamic inputs mainly in layer IV [9]. In contrast, the neural circuits mediated through the corpus callosum originate mainly from pyramidal neurons located in layers II/III and V [10] and terminate mainly in layer II/III of the opposite side [11, 12]. Therefore, the reconstructed activities in the S1 of the opposite side are expected to have a unique depth and temporal profiles that are different from those of the activities in the S1 of untreated mice. However, we cannot investigate the expected properties, because the depth profile of cortical activities cannot be observed with the conventional optical imaging technique used in our previous study [8]. Furthermore, the time course of fluorescence signals is obscured by activity-dependent hemodynamic responses on the cortical surface [13]. Recently, however, a macroconfocal microscope has been developed [14], which may be applicable for tomographic optical imaging of the reconstructed activities in the S1 opposite to the injury. In the present study, we tested this possibility.

## Materials and methods

The present study was performed according to the guidelines for animal experiments of Niigata University and was approved by the ethics committee of Niigata University. Male C57BL/6 mice were used in all experiments.

### Crossing nerve transfer

Mice at 8 weeks of age were anesthetized with pentobarbital (40 mg/kg, i.p.). Crossing nerve transfer was performed, as described previously [8]. The BPs of both sides were exposed under sterile conditions. The medial cord, median nerve, and ulnar nerve were identified under a binocular microscope. On the recipient side, the right medial cord was cut at the level just proximal to the point where the medial cord diverges into the median and ulnar nerves. The remaining left radial and musculocutaneous nerves were cut because left forepaw stimulation produces cortical responses in the contralateral S1 via these nerves [15]. On the donor side, the left medial cord was cut at the level distal to the pectoralis branch origin. A sciatic nerve graft (approximately 2 cm long) was taken from another mouse anesthetized with pentobarbital. The cut ends of the nerves were connected to the nerve graft with 11–0 sutures. The nerve graft and BPs were covered by suturing the skin. Fradiomycin (Mochida Pharmaceutical, Tokyo, Japan) and ampicillin (Meiji Seika, Tokyo, Japan) were used to prevent infections.

### Conventional surface optical imaging of cortical responses

Imaging experiments were performed as described previously [8]. Mice were anesthetized with urethane (1.7 g/kg, i.p.), at 16 weeks of age for the untreated mice and at 8 weeks after crossing nerve transfer in the operated mice. Throughout the recordings, the rectal temperature was maintained at 38°C using a silicon rubber heater. The surgical procedures were conducted under sterile conditions. After subcutaneous injection of bupivacaine (AstraZeneca, Osaka, Japan), the disinfected skin was incised, and the portion of the skull covering the imaged area was exposed. The surface of the skull was cleaned with sterile saline, and a small piece of metal was attached to the skull with dental acrylic resin (Super Bond; Sun Medical, Shiga, Japan). The piece of metal was screwed to a manipulator to fix the head position. The skull was covered with a mixture of liquid paraffin and Vaseline to prevent it from drying and to keep it transparent. Imaging was started approximately 60 min after the administration of urethane. An additional dose of urethane (0.2 g/kg, s.c.) was administered when necessary.

Cortical images (128×168 pixels or 4.6×6.0 mm) of green fluorescence (λ = 500–550 nm) in blue excitation light (λ = 450–490 nm) were recorded using a cooled CCD camera system (AQUACOSMOS/Ratio system with an ORCA-ER camera; Hamamatsu Photonics, Hamamatsu, Japan). The camera was attached to a binocular epifluorescence microscope (MZ FL III; Leica Microsystems, Wetzlar, Germany) with an objective lens (magnification: 1.0, numerical aperture: 0.125). Cortical responses were elicited by sinusoidal vibration (displacement: ±0.4 mm, 50 Hz for 0.5 s), which was produced with a mechanical stimulator (DPS-270; Dia Medical, Tokyo, Japan), and applied with a small brush to the left plantar forepaw. Images were obtained at 9 frames/s, which were averaged over 24 or 48 trials. Moving spatial averaging in 5×5 pixels was used for smoothing and improving the image quality. The normalized images in ΔF/F_0_ were shown in a pseudocolor scale, in which ΔF and F_0_ were the increase in fluorescence intensity and averaged intensity in five frames immediately before to stimulation, respectively. The response amplitude in ΔF/F_0_ was evaluated in a square window of 10×10 pixels (0.36×0.36 mm). The window location was adjusted, so that the response amplitude in the window was maximal. To estimate the extent of bilateral cortical representation in S1, the bilaterality index was calculated as the ratio of response amplitudes in both hemispheres. In the untreated mice, the bilaterality index was defined as the response amplitudes in the left S1 normalized to those in the right S1. In the operated mice, the index was defined as the response amplitudes in the right S1 normalized to those in the left S1. At the end of imaging experiments, mice were euthanized with an overdose of pentobarbital (i.p.).

### Tomographic optical imaging of cortical responses

Tomographic imaging of cortical responses in the right S1 was performed in the untreated and operated mice. The surgical procedures were similar to those used in conventional surface imaging, except that the skull covering the imaged area was thinned, covered with 2% agarose (Type I-B; Sigma-Aldrich, St. Louis, U.S.A.) dissolved in saline, and sealed with a cover glass. For imaging, we used a macroconfocal microscope (D-Eclipse C1 system, Nikon, Tokyo, Japan) with an objective lens (AZ Plan Fluor, magnification: 5.0×, numerical aperture: 0.5, working distance: 15 mm), which was combined with a zoom magnification of 1.6×. A pinhole of maximal size (diameter: 150 μm) was used. The excitation light was obtained from a blue laser at λ = 488 nm, and the green fluorescence (λ = 500–530 nm) was detected. At first, the cortical surface was identified based on the fluorescence images, and the tomographic imaging planes were set at 50, 200, 400 and 800 μm from the cortical surface. The sensitivity of the photodetector was adjusted so that the original images were neither saturated nor too dark. Serial images with a resolution of 256×256 pixels (2.6×2.6 mm) were taken at a frame rate of 400 ms per frame. This trial was repeated at 50 s intervals for 48 times at each depth. The obtained images were saved in TIFF format, and processed by the same AQUACOSMOS software used for conventional surface imaging. The amplitude and time course of the response was measured from the rate of fluorescence changes (ΔF/F_0_) in a circular window with a diameter of 50 pixels (0.51 mm).

### Statistical analysis

Statistical significance was evaluated by using the Mann-Whitney U test. Only significant differences (P<0.05) were shown in the figures. The number (n) in each figure represents that of mice used in the experiment.

## Results

### Conventional surface optical imaging of cortical responses after crossing nerve transfer

**Fig 1:**
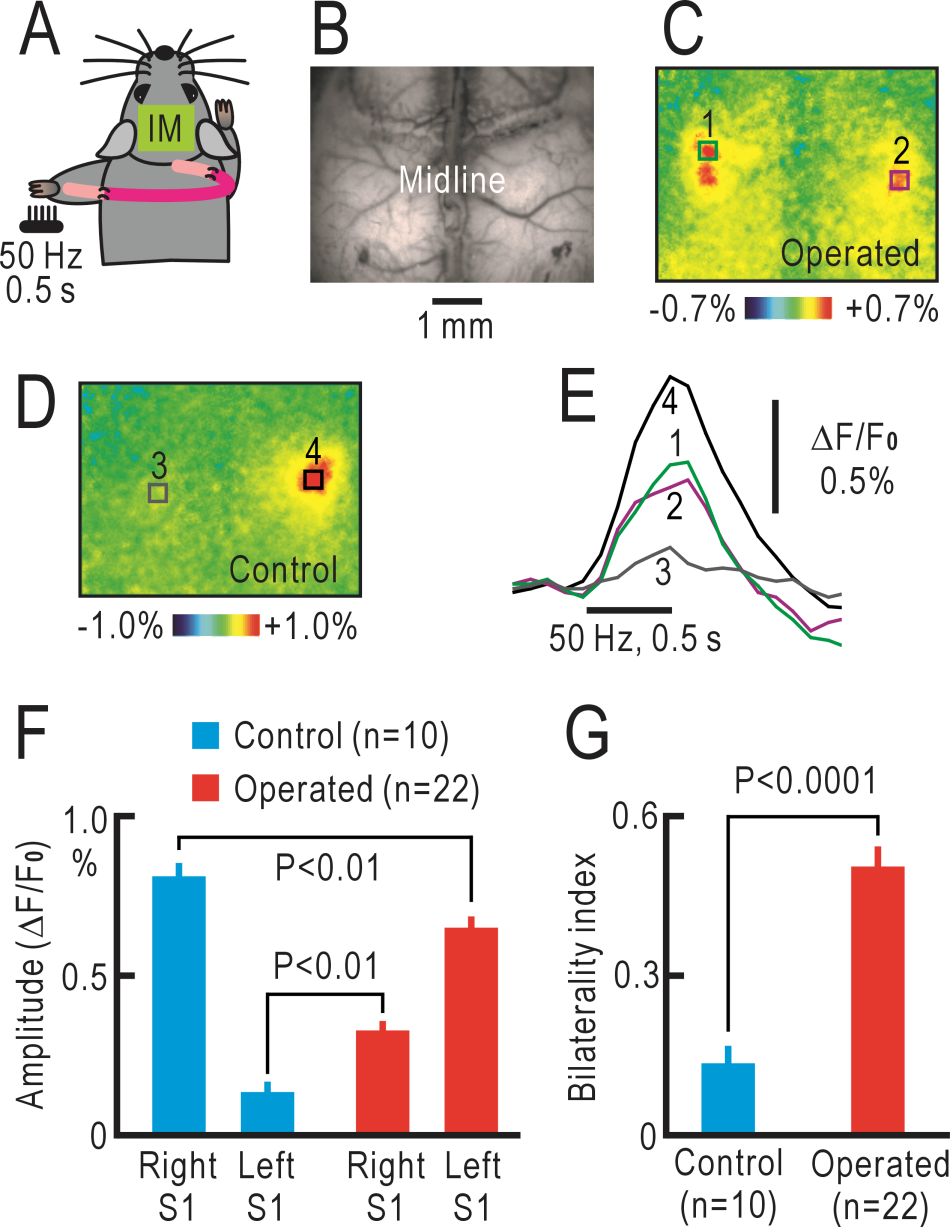
Conventional imaging of cortical responses after crossing nerve transfer. (A) Schematic drawing of crossing nerve transfer. IM represents imaged area. (B) Original fluorescence image. (C) Pseudo-color image of fluorescence responses elicited by vibratory stimulation at 50 Hz for 0.5 s applied to the left forepaw. The image in (B) and the responses in (C) were recorded in the same mouse with crossing nerve transfer. (D) Fluorescence responses elicited by stimulation of the left forepaw in an untreated mouse. The contralateral right S1 shows clear responses, while the ipsilateral left S1 is only weakly activated. (E) Time course of ΔF/F_0_ changes in the square windows (1–4) shown in (C) and (D). (F) Amplitudes of the cortical responses. Mean and S.E.M. are shown. (G) Bilaterality index (ratio of the inferior response amplitudes normalized by the superior response amplitudes) in the untreated and operated mice.

In the operated mice, the medial cord containing the median and ulnar nerves from the left forepaw were cut, and the distal cut end was sutured to the central cut end of the right medial cord using a sciatic nerve graft obtained from another mouse (Fig 1A). We investigated the S1 responses 8 weeks after the operation by conventional surface imaging of flavoprotein fluorescence signals (Fig 1B). When the left forepaw was stimulated with brush vibration (50 Hz for 0.5 s), both the right and left S1 showed fluorescence responses (Fig 1C). However, in the untreated mice, the left S1 exhibited only weak responses, whereas the responses in the right S1 were very clear (Fig 1D). The four types of cortical responses peaked approximately at 0.5–0.6 s after the stimulus onset, and no obvious difference was found between the time courses of the responses (Fig 1E). However, when comparing the superior responses (responses in the left S1 in the operated mice and those in the right S1 in the untreated mice), the amplitudes in the untreated group were significantly higher than those in the operated mice (P<0.01, Fig 1F), whereas the inferior responses in the untreated group were significantly lower than those in the operated mice (P<0.01). These differences were clearly demonstrated by the bilaterality index, which was significantly different between the untreated and operated mice (P<0.0001, Fig 1F). These findings clearly reproduced our previous results [8].

### Tomographic optical imaging of cortical responses in untreated mice

**Fig 2:**
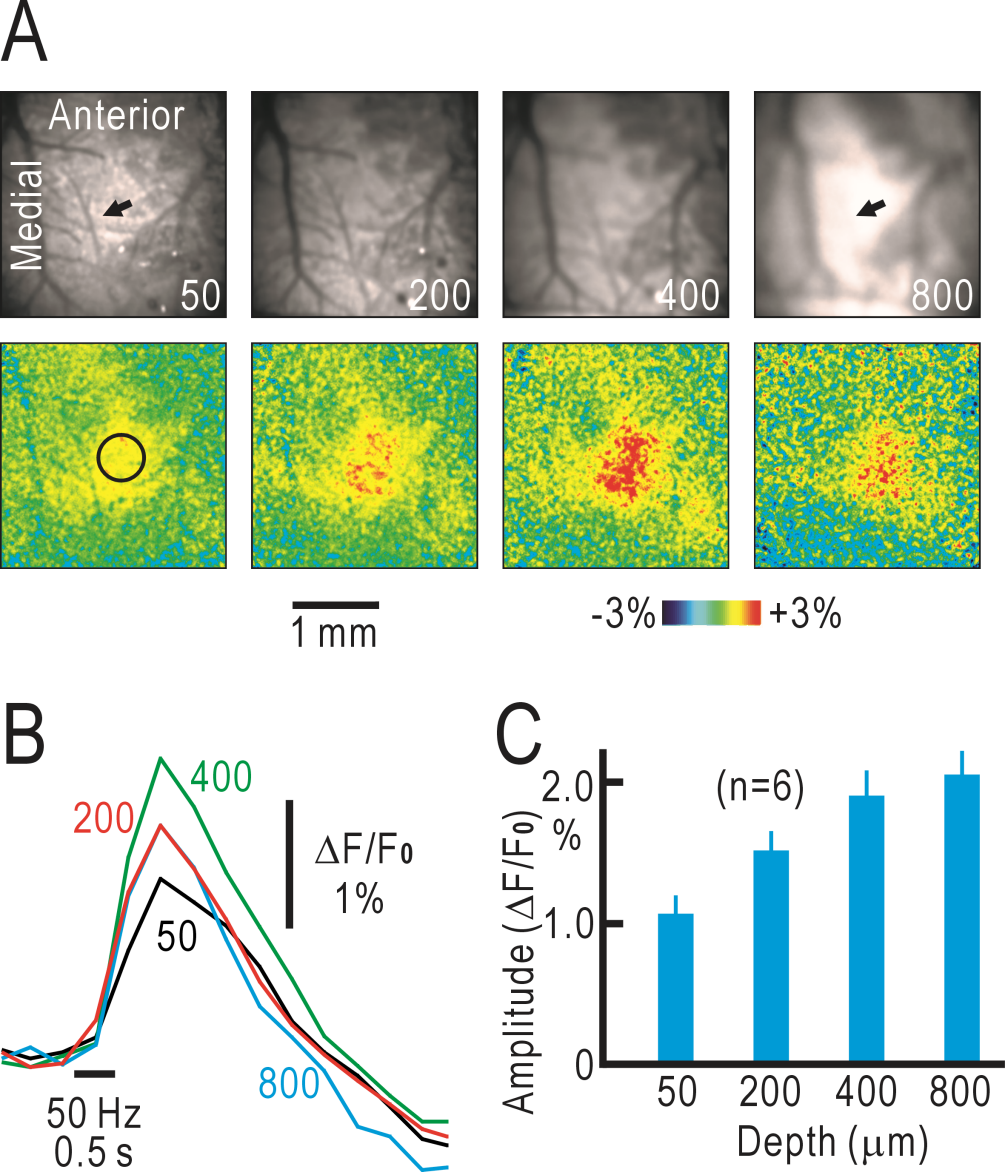
Tomographic imaging of cortical responses in untreated mice. (A) Original tomographic images in the right S1 (upper panels) and pseudo-color images of fluorescence responses elicited by vibratory stimulation at 50 Hz for 0.5 s applied to the left forepaw (lower panels). The numbers in the upper panels represent the depth (μm) from the cortical surface. The two arrows in the upper panels show the position of an artery that is visible in the leftmost panel but not in the rightmost panel. The circle in the leftmost lower panel shows the circular window in which response amplitudes were measured in ΔF/F_0_. (B) Time courses of the fluorescence responses measured at 50, 200, 400 and 800 μm deep from the cortical surface using a macroconfocal microscope. Data in (A) and (B) were obtained from the same mouse. (C) Amplitudes of fluorescence responses measured at each depth.

We investigated the dominant responses of the right S1 to left forepaw stimulation in untreated mice by macroconfocal microscopy. The tomographic image plane was set at 50 μm, 200 μm, 400 μm and 800 μm from the cortical surface, thus approximately corresponding to the depth of layer I, layer II/III, layer IV, and layer V/VI, respectively [16]. In the original fluorescence images, the blood vessels observed at 50 μm became blurred in the deeper layers (upper panels of Fig 2A). Furthermore, the arteriole observed at 50 μm (arrow in the leftmost upper panel) was not found at 800 μm (arrow in the rightmost upper panel), indicating that tomographic effects could be achieved by macroconfocal microscopy. As for the cortical responses to forepaw stimulations, the fluorescence increases shown in each lower panel were observed at approximately the same horizontal position, suggesting that a columnar structure was preserved in the somatosensory responses elicited by forepaw stimulation (Fig 2A). Changes in ΔF/F_0_ peaked at approximately 0.9 s after the stimulus onset (Fig 2B), and no obvious time course difference was observed between the responses in each layer. In the macroconfocal imaging, the frame rate (400 ms per frame) was much slower than that of the conventional surface imaging (111 ms per frame). Even so, the time course of the responses (for example, Fig 2B) seemed to be slower than the time course of the fluorescence response observed at the cortical surface (for example, Fig 1E). The response amplitudes at 50 μm and 200 μm were smaller than those at 400 μm and 800 μm. These results indicated that tomographic flavoprotein fluorescence imaging of the cortical responses can be performed by macroconfocal microscopy, revealing a slower response time course than that observed on the brain surface. In tomographic imaging, the response amplitudes at layer I were smaller than those at the other layers, while the signals at layer IV and deeper layers might not be detected efficiently in conventional imaging. Therefore, it is likely that the contribution of layer II/III is the largest in the cortical responses recorded by conventional imaging (for example, Fig 1).

### Tomographic optical imaging of cortical responses in the operated mice

**Fig 3:**
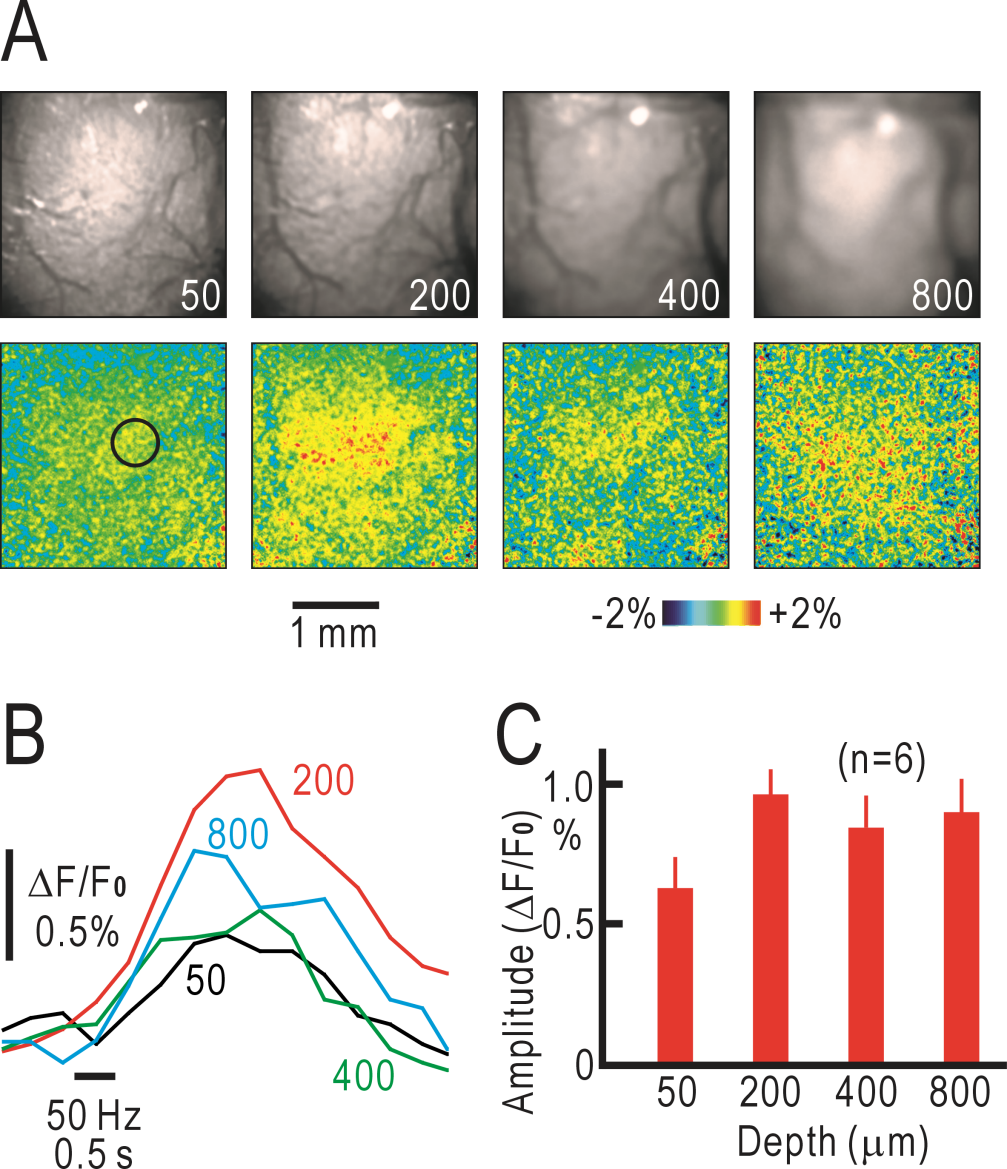
Tomographic imaging of cortical responses in operated mice. (A) Original tomographic images in the right S1 (upper panels) and pseudocolor images of fluorescence responses elicited by vibratory stimulation at 50 Hz for 0.5 s applied to the left forepaw (lower panels). (B) Time courses of the fluorescence responses measured at 50, 200, 400 and 800 μm deep from the cortical surface using a macroconfocal microscope. Data in (A) and (B) were obtained from the same mouse. (C) Amplitudes of fluorescence responses measured at each depth.

We observed the responses of the right S1 to left forepaw stimulation in the operated mice by macroconfocal microscopy. The spatial distribution of the fluorescence responses was more diffuse than that in the untreated mice and slightly different between different imaging planes, suggesting that the functional columnar structure was deteriorated after the operation (lower panels in Fig 3A). The time course of the fluorescence responses was much slower compared to that in the untreated mice at any depth, and showed a peak at approximately 1.7 s after stimulus onset (Fig 3B). The response amplitudes at 50 μm were smaller than those at other depths, whereas the amplitudes at 200 μm were larger than those at 400 μm and 800 μm (Fig 3B). Taken together, these results indicate that the reconstructed responses after crossing nerve transfer showed a deteriorated columnar structure, were smaller in amplitude, and were slower in time course compared with the cortical responses in untreated mice. Furthermore, the responses in layer II/III seemed to be more dominant compared to the responses in the other layers.

### Comparison of tomographic cortical responses between untreated and operated mice

**Fig 4:**
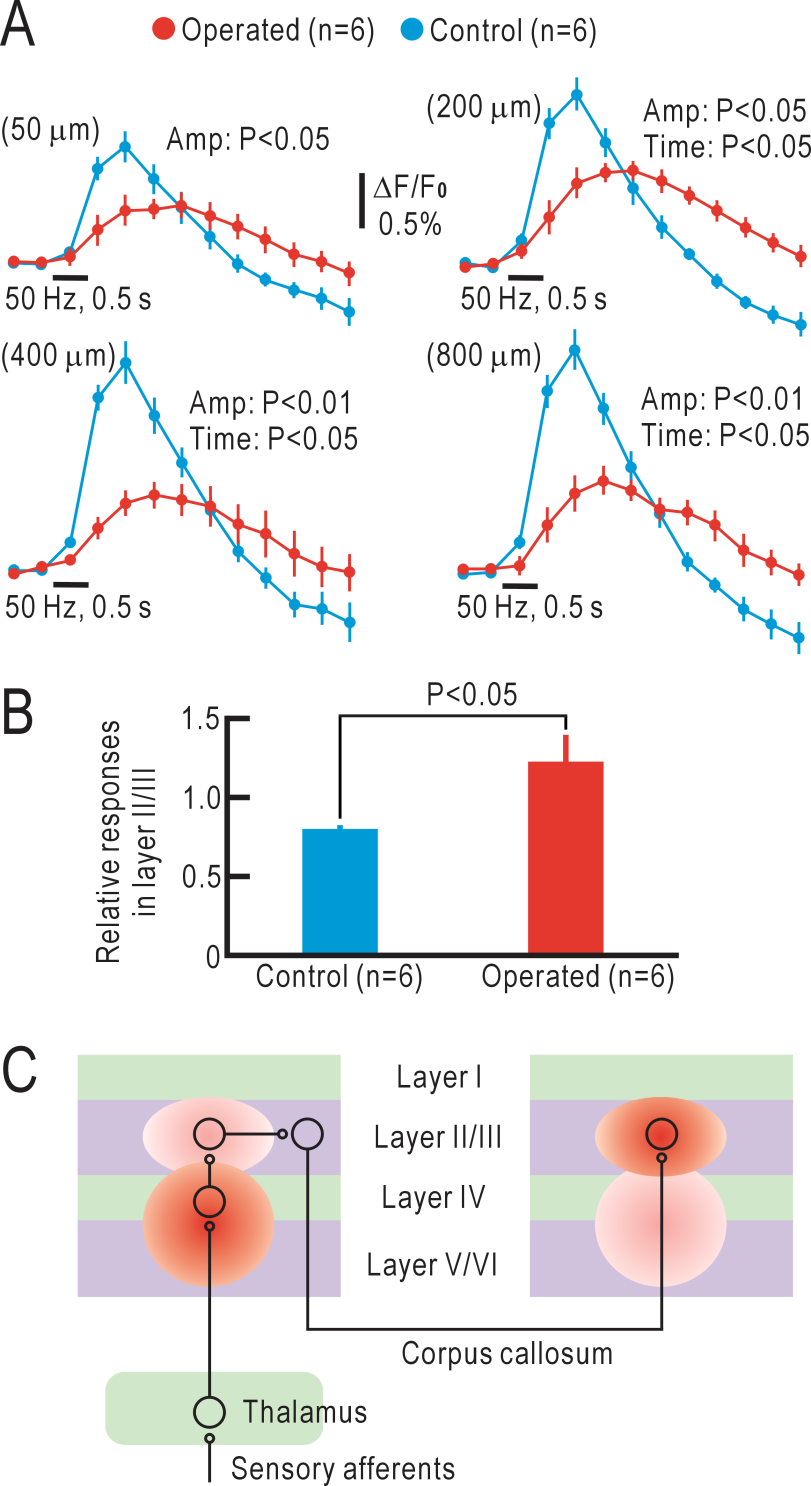
Comparison of cortical responses between untreated and operated mice. (A) Time courses of the fluorescence responses measured at 50, 200, 400 and 800 μm deep from the cortical surface using a macroconfocal microscope. Statistical differences were evaluated regarding the peak amplitude and peak latency between the untreated and operated mice. (B) Relative response amplitudes at 200 μm normalized to those at 400 μm. This ratio is smaller than 1.0 in the untreated mice, while it was larger than 1.0 in the operated mice. (C) Schematic drawing of the neural circuits in the operated mice.

The time course of somatosensory responses was compared between the untreated and operated mice (Fig 4A). The peak amplitude was significantly greater in the untreated group at any depth (P<0.05 at 50 μm and 200 μm, P<0.01 at 400 μm and 800 μm). The peak latency was faster in the untreated group at any depth, but only the values at 200 μm, 400 μm, and 800 μm showed a significant difference (P<0.05, respectively). To analyze the quantitative distribution of the response amplitude in the depth direction, the relative response amplitudes at 200 μm normalized to those at 400 μm were calculated for each mouse and compared between the two groups. In the untreated group, the relative amplitude at 200 μm was less than 1.0 (amplitude at 200 μm < amplitude at 400 μm), whereas it was greater than 1.0 (amplitude at 200 μm > amplitude at 400 μm) in the operated group, with a significant group difference (P<0.05, Fig 4B). These results support the hypothesis that the cortical responses in the right S1 of the untreated mice may be directly driven by the thalamic afferents that terminate in layer IV, whereas the responses in the right S1 of the operated mice are likely driven by the callosal afferents that terminate in layer II/III (Fig 4C).

## Discussion

In the present study, we used endogenous green fluorescence signals derived from mitochondrial flavoproteins [17] to investigate cortical reorganization after crossing nerve transfer. Endogenous signals are weaker than fluorescence signals derived from artificial fluorophores. However, imaging techniques based on endogenous signals are devoid of any artificial effects of fluorophores and can be applicable for investigating various types of cortical plasticity [15, 18, 19] or cortical reorganization [8, 20, 21]. Usually, two-photon microscopy is used for tomographic imaging of cortical activities [22, 23]. Flavoprotein fluorescence signals have been used for static imaging with two-photon microscopes in previous studies [24–26]. We attempted to visualize the dynamic cortical responses with tomographic imaging of flavoprotein fluorescence signals by two-photon microscopy. However, the signals were too weak to visualize cortical activities, which might be because of the low quantal efficiency of flavoproteins as fluorophores. Macroconfocal microscopy is an alternative method for tomographic imaging based on flavoprotein fluorescence [14]. However, there are a number of technical problems in this method. First, the depth resolution is critically dependent on magnification (S1A and S1B Fig). Furthermore, fluorescence originated from deeper layers was scattered by brain tissue. Therefore, magnification must be lowered to reduce depth resolution and the pin hole must be maximized, so that the fluorescence from deeper layers can pass through the pin hole. As a result, spatial resolution at neuronal level is difficult to be achieved. The sensitivity of the photodetector must be adjusted to visualize fine structures as much as possible. As a result, the images of surface vessels were visible even when the focal place was set at deeper cortical layers (for example, Figs 2A and 3A). However, activities in supragranular, granular and infragranular layers should be differentiated even under a low depth resolution around 100–200 μm in the present experimental conditions (S1A and S1B Fig). The complex light scattering properties of the cerebral cortex are difficult to reproduce. Therefore, the data in the present study are not quantitative regarding the response distribution in each layer. However, we performed a model experiment using 1% soybean oil emulsion as a scattering medium instead of brain tissue. The results indicated that shapes such as letter “A” were visible using our macroconfocal microscope through the scattering medium with a thickness of 600 μm but not 900 μm (S1C Fig). Therefore, the imaging data taken at 400 μm from the cortical surface are likely to reflect neural activities at the depth, while those taken at 800 μm may reflect scattered signals originating from more superficial layers. The present results demonstrated that the layer-specific response properties were modified by crossing nerve transfer. These results are compatible with the expectation that tomographic imaging of cortical responses may be practical when flavoprotein fluorescence is excited by a single-photon and visualized by macroconfocal microscopy.

Previously, we have reported that cortical responses were induced in the S1 opposite to the stimulated forepaw 8 weeks after crossing nerve transfer [8]. We reproduced these results in the present study (Fig 1). We assumed that the observed responses were induced by propagation of neural activities from the ipsilateral S1 to the contralateral S1 through the corpus callosum. However, there were some unresolved points regarding this interpretation. First, the corpus callosum is thought to terminate mainly at layer II/III [10, 11], whereas the sensory afferents terminate mainly at layer IV [9]. Therefore, there are two possibilities for our observations: (1) the callosal inputs may drive layer IV in the operated mice, or (2) the reorganized responses may be initiated in layer II/III to drive responses in other layers. In the present study, the cortical responses in layer II/III of the operated mice were dominant compared with those in layer IV, thus, supporting the latter possibility. Second, the time course of the reorganized cortical responses should be slower than the original cortical responses in time course, although no such difference was found in the cortical responses observed on the cortical surface (for example, Fig 1E). In tomographic imaging, however, the time course of the reorganized cortical responses in the operated mice was clearly slower than that of the original cortical responses in the untreated mice (Fig 4A). One possible explanation for this discrepancy is that the flavoprotein fluorescence signals observed by macroconfocal microscopy are less affected by surface arterioles, in which hemodynamic responses are induced by neural activities [13, 27]. Hemodynamic responses are induced in penetrating arterioles as well as in surface arterioles [28]. However, the optical images of penetrating arterioles, observed as dots from the cortical surface, are much smaller than those of surface arterioles. Therefore, the optical effects of the former responses are expected to be smaller than the latter responses. The time course of flavoprotein fluorescence signals recorded in brain slices is known to be much slower than that recorded *in vivo* [17]. This discrepancy may be partly explained by the artificial effects of hemodynamic responses in conventional imaging. At present, we cannot exclude other possibilities that may explain the difference in time course of recorded responses between the two imaging methods. However, we believe that the time course in macroconfocal imaging is more realistic compared with that in conventional surface imaging. Interestingly, positive macroconfocal responses in the control mice were followed by a slow negative phase, while no negative phase was found in the operated mice (Fig 4A). Negative responses can be explained by hemodynamic responses or net neural inhibition. Since fluorescence signals recorded in macroconfocal imaging are not strongly affected by hemodynamic responses, the difference in Fig 4A may be explained by the presence or absence of neural inhibition. In accordance with this view, negative fluorescence responses elicited by neural inhibition are sometimes observed immediately after neural stimulation [29], a period before hemodynamic responses are initiated. Taken together, these findings indicate that tomographic imaging by macroconfocal microscopy clearly demonstrates the reorganized cortical responses after crossing nerve transfer in mice.

As discussed in our previous paper [8], avulsion of nerve roots in the BP is repaired by various surgical operations including nerve transfer between the injured nerve ends and the accessory or intercostal nerves [30] or the phrenic nerves [31]. The somatosensory system can be drastically reorganized after peripheral injuries [32–34]. After such operations, however, new pathways must be produced for functional recovery with potential rewiring errors [35]. The rewiring error could be minimal in crossing nerve transfer, because neurite extensions from the ipsilateral sensory pathways and reinforcement of the existing callosal fibers are sufficient for the restoration of cortical activities contralateral to the injured side. Importance of callosal fibers in reorganization of the somatosensory system after crossing nerve transfer has been clearly demonstrated by the dissection of the corpus callosum performed in our previous study [8]. Callosal pathways that connect both sides of the cortex play important roles in sensory information transfer between both sides of S1 corresponding to body parts, in which bilateral coordination is essential [36–38] as well as in midline areas, such as the intraoral cavity, chin, or trunk [39]. In the visual system, retinal image reversal is easily achieved by using prism spectacles, and reversed visual inputs induce adaptation to reversed vision together with bilateral cortical representation in the visual cortex [40–42]. Therefore, crossing nerve transfer could also result in functional adaptation to reversed somatosensory inputs. Crossing nerve transfer in a mouse model confirms the clinical results on the recovery in sensory functions, and some aspects of clinical time courses in operated mice have been described in our previous paper [8]. However, the present results also suggested certain limitations of crossing nerve transfer, such as slow time course and disrupted functional columnar and layer structures in the reorganized cortical responses contralateral to the repaired hand. Using these detailed parameters of the cortical responses as indicators of functional recovery, it might be possible to compare or evaluate various surgical treatments or rehabilitation methods for avulsion injuries to the BP at the experimental level [43, 44].

## Supporting information

S1 Fig Depth resolution of macroconfocal system. (A) Images of letter “A” taken at the various depths of the focal plane. The sensitivity of the photodetector was fixed in this experiment. A 5× objective lens combined with a zoom magnification (1.6×) was used. (B) Relative intensity of images at the various depths of the focal plane. The zoom magnification was changed between 1.2× and 2.0×. (C) Images of letter “A” taken through 1% soybean oil emulsion (Intralipos, Otsuka, Tokyo, Japan) of a thickness between 0 and 900 μm.
(TIF)
