# Supplementary figures and images for: Tomographic optical imaging of cortical responses after crossing nerve transfer in mice
Source: PLoS One. 2018 Feb 14;13(2):e0193017. doi: 10.1371/journal.pone.0193017 (PMC5812646; doi:10.1371/journal.pone.0193017)

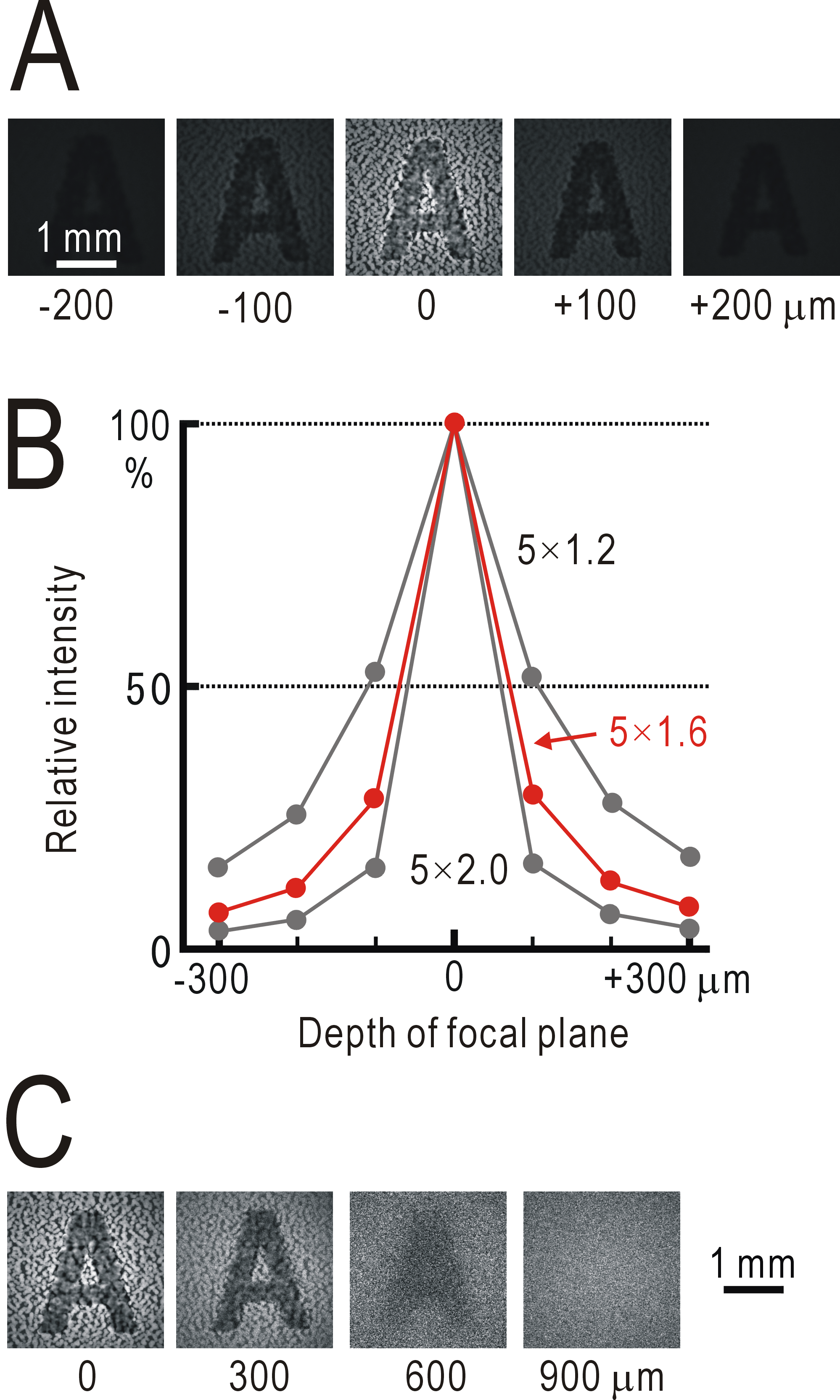

Supplement: S1 Fig — (A) Images of letter “A” taken at the various depths of the focal plane. The sensitivity of the photodetector was fixed in this experiment. A 5× objective lens combined with a zoom magnification (1.6×) was used. (B) Relative intensity of images at the various depths of the focal plane. The zoom magnification was changed between 1.2× and 2.0×. (C) Images of letter “A” taken through 1% soybean oil emulsion (Intralipos, Otsuka, Tokyo, Japan) of a thickness between 0 and 900 μm. (TIF) [file pone.0193017.s001.tif]
